# Supplementary material for: Characterization of Phosphorylation Status and Kinase Activity of Src Family Kinases Expressed in Cell-Based and Cell-Free Protein Expression Systems
Source: Biomolecules. 2021 Oct 2;11(10):1448. doi: 10.3390/biom11101448 (PMC8533471; doi:10.3390/biom11101448)
Supplement: Supplementary file 1 [file biomolecules-11-01448-s001.zip › biomolecules-1381980-supplementary.pdf]

biomolecules

MDPI

Supplementary Information S1

Alignment of eight SFKsequences by ClustalW analysis. Phosphorylation site by CSK and autophosphorylation site in kinase domain are shown in bold.

```
P12931|SRC_HUMAN      ---------------------MGSNKSKP-KDASQRRRSLEPAENVHGAG 28
P07947|YES_HUMAN      ---------------------MGCIKSKENKSPAIKYRPENTPEPVSTSV 29
P09769|FGR_HUMAN      ---------------------MGCVFCKKLEPVATAKEDAGLEGDFRSYG 29
P06241|FYN_HUMAN      ---------------------MGCVQCKDKEATKLTEERDGSLNQSSGY- 28
P08631|HCK_HUMAN      MGGRSSCEDPGCPRDEERAPRMGCMKSFKLQVGGN--TFSKTETSASPHC 48
P07948|LYN_HUMAN      ---------------------MGCIKSKGKDSLSDDGVDLKTQPVRNTER 29
P06239|LCK_HUMAN      ---------------------MGCGCS----SHPEDDWMENIDVCENCHY 25
P51451|BLK_HUMAN      ---------------------MGLVSSK---KPDKEKPIKEKDKGQWSPL 26

P12931|SRC_HUMAN      G--GAFPASQTPSKPASADGHRG--PSAAFAPAAAEP--KLFGGFNSSDT 72
P07947|YES_HUMAN      SHYGAEPTTVSPCPSSSAKGTAVNFSSLSMTPFGGSSGVTPFGGASSSFS 79
P09769|FGR_HUMAN      A---ADHYGPDPT-KARPASSFAHIPNYSNFSSQ-----AINPGFLDS-- 68
P06241|FYN_HUMAN      ------RYGTDPTPQHYPSFGVTSIPNYNNFHAAGGQGLTVFGGVNSSSH 72
P08631|HCK_HUMAN      P-----VYVPDPTSTIKPGPNSH---------------------NSNTPG 72
P07948|LYN_HUMAN      T-----IYVRDPTSNKQQRPVPE---------------------SQLLPG 53
P06239|LCK_HUMAN      P-----IVPLDGKGTLLIRNGSE---------------------VRDPLV 49
P51451|BLK_HUMAN      K-----VSAQDKDAPPLPPLVVF---------------------NHLTPP 50

P12931|SRC_HUMAN      VTSPQRAGPLAGGVTTFVALYDYESRTETDLSFKKGERLQIVNNTEGDWW 122
P07947|YES_HUMAN      VVPSSYPAGLTGGVTIFVALYDYEARTTEDLSFKKGERFQIINNTEGDWW 129
P09769|FGR_HUMAN      -GTIRGVSGIG--VTLFIALYDYEARTEDDLTFTKGEKFHILNNTEGDWW 115
P06241|FYN_HUMAN      TGTLRTRGGTG--VTLFVALYDYEARTEDDLSFHKGEKFQILNSSEGDWW 120
P08631|HCK_HUMAN      ----IREAGS--EDIIVVALYDYEAIHHEDLSFQKGDQMVVLEES-GEWW 115
P07948|LYN_HUMAN      QRFQTKDPEE--QGDIVVALYPYDGIHPDDLSFKKGEKMKVLEEH-GEWW 100
P06239|LCK_HUMAN      TYEGSNPPASPLQDNLVIALHSYEPSHDGDLGFEKGEQLRILEQS-GEWW 98
P51451|BLK_HUMAN      PPDEHLDEDK----HFVVALYDYTAMNDRDLQMLKGEKLQVLKGT-GDWW 95

P12931|SRC_HUMAN      LAHSLSTGQTGYIPSNYVAPSDSIQAEEWYFGKITRRESERLLLNAENPR 172
P07947|YES_HUMAN      EARSIATGKNGYIPSNYVAPADSIQAEEWYFGKMGRKDAERLLLNPGNQR 179
P09769|FGR_HUMAN      EARSLSSGKTGCIPSNYVAPVDSIQAEEWYFGKIGRKDAERQLLSPGNPQ 165
P06241|FYN_HUMAN      EARSLTTGETGYIPSNYVAPVDSIQAEEWYFGKLGRKDAERQLLSFGNPR 170
P08631|HCK_HUMAN      KARSLATRKEGYIPSNYVARVDSLETEEWFFKGISRKDAERQLLAPGNML 165
P07948|LYN_HUMAN      KAKSLLTKKEGFIPSNYVAKLNTLETEEWFFKDITRKDAERQLLAPGNSA 150
P06239|LCK_HUMAN      KAQSLTTGQEGFIPFNFVAKANSLEPEPWFFKNLSRKDAERQLLAPGNTH 148
P51451|BLK_HUMAN      LARSLVTGREGYVPSNFVARVESLEMERWFFRSQGRKEAERQLLAPINKA 145

P12931|SRC_HUMAN      GTFLVRESETTKGAYCLSVSDFDNAKGLNVKHYKIRKLDSGGFYITSRTQ 222
P07947|YES_HUMAN      GIFLVRESETTKGAYSLSIRDWDEIRGDNVKHYKIRKLDNGGYYITTRAQ 229
P09769|FGR_HUMAN      GAFLIRESETTKGAYSLSIRDWDQTRGDHVKHYKIRKLDMGGYYITTRVQ 215
P06241|FYN_HUMAN      GTFLIRESETTKGAYSLSIRDWDDMKGDHVKHYKIRKLDNGGYYITTRAQ 220
P08631|HCK_HUMAN      GSFMIRDSETTKGSYSLSVRDYDPRQGDTVKHYKIRTLDNGGFYISPRST 215
P07948|LYN_HUMAN      GAFLIRESETLKGSFSLSVRDFDPVHGDVIKHYKIRSLDNGGYYISPRIT 200
P06239|LCK_HUMAN      GSFLIRESESTAGSFSLSVRDFDQNQGEVVKHYKIRNLDNGGFYISPRIT 198
P51451|BLK_HUMAN      GSFLIRESETNKGAFSLSVKDVT-TQGELIKHYKIRCLDEGGYYISPRIT 194

P12931|SRC_HUMAN      FNSLQQLVAYYSKHADGLCHRLTTVCPTSKPQTQGL---AKDAWEIPRES 269
P07947|YES_HUMAN      FDTLQKLVKHYTEHADGLCHKLTTVCPTVKPQTQGL---AKDAWEIPRES 276
P09769|FGR_HUMAN      FNSVQELVQHYMEVNDGLCNLLIAPCTIMKPQTLGL---AKDAWEISRSS 262
P06241|FYN_HUMAN      FETLQQLVQHYSERAAGLCCRLVVPCHKGMPRLTDLSVKTKDVWEIPRES 270
P08631|HCK_HUMAN      FSTLQELVDHYKKGNDGLCQKLSVPCMSSKPQKPWE----KDAWEIPRES 261
P07948|LYN_HUMAN      FPCISDMIKHYQKQADGLCRRLEKACISPKPQKPWD----KDAWEIPRES 246
P06239|LCK_HUMAN      FPGLHELVRHYTNASDGLCTRLSRPCQTQKPQKPWW----EDEWEVPRET 244
P51451|BLK_HUMAN      FPSLQALVQHYSKKGDGLCQRLTLPCVRPAPQNPWA----QDEWEIPRQS 240
```

```
P12931|SRC_HUMAN     LRLEVKLGQGCFGEVWMGTWNGTTRVAIKTLKPGTMSPEAFLQEAQVMKK 319
P07947|YES_HUMAN     LRLEVKLGQGCFGEVWMGTWNGTTKVAIKTLKPGTMMPEAFLQEAQIMKK 326
P09769|FGR_HUMAN     ITLERRLGTGCFGDVWLGTWNGSTKVAVKTLKPGTMSPKAFLEEAQVMKL 312
P06241|FYN_HUMAN     LQLIKRLGNGQFGEVWMGTWNGNTKVAIKTLKPGTMSPESFLEEAQIMKK 320
P08631|HCK_HUMAN     LKLEKKLGAGQFGEVWMATYNKHTKVAVKTMKPGSMSVEAFLAEANVMKT 311
P07948|LYN_HUMAN     IKLVKRLGAGQFGEVWMGYYNNSTKVAVKTLKPGTMSVQAFLEEANLMKT 296
P06239|LCK_HUMAN     LKLVERLGAGQFGEVWMGYYNGHTKVAVKSLKQGSMSPDAFLAEANLMKQ 294
P51451|BLK_HUMAN     LRLVRKLGSGQFGEVWMGYYKNNMKVAIKTLKEGTMSPEAFLGEANVMKA 290

P12931|SRC_HUMAN     LRHEKLVQLYAVVSE-EPIYIVTEYMSKGSLLDFLKGETGKYLRLPQLVD 368
P07947|YES_HUMAN     LRHDKLVPLYAVVSE-EPIYIVTEFMSKGSLLDFLKEGDGKYLKLPQLVD 375
P09769|FGR_HUMAN     LRHDKLVQLYAVVSE-EPIYIVTEFMCHGSLLDFLKNPEGQDLRLPQLVD 361
P06241|FYN_HUMAN     LKHDKLVQLYAVVSE-EPIYIVTEYMNKGSLLDFLKDGEGRALKLPNLVD 369
P08631|HCK_HUMAN     LQHDKLVKLHAVVTK-EPIYIITEFMAKGSLLDFLKSDEGSKQPLPKLID 360
P07948|LYN_HUMAN     LQHDKLVRLYAVVTREEPIYIITEYMAKGSLLDFLKSDEGGKVLLPKLID 346
P06239|LCK_HUMAN     LQHQRLVRLYAVVTQ-EPIYIITEYMENGSLVDFLKTPSGIKLTINKLLD 343
P51451|BLK_HUMAN     LQHERLVRLYAVVTK-EPIYIVTEYMARGCLLDFLKTDEGSRLSLPRLID 339

P12931|SRC_HUMAN     MAAQIASGMAYVERMNYVHRDLRAANILVGENLVCKVADFGLARLIEDNE 418
P07947|YES_HUMAN     MAAQIADGMAYIERMNYIHRDLRAANILVGENLVCKIADFGLARLIEDNE 425
P09769|FGR_HUMAN     MAAQVAEGMAYMERMNYIHRDLRAANILVGERLACKIADFGLARLIKDDE 411
P06241|FYN_HUMAN     MAAQVAAGMAYIERMNYIHRDLRSANILVGNGLICKIADFGLARLIEDNE 419
P08631|HCK_HUMAN     FSAQIAEGMAFIEQRNYIHRDLRAANILVSASLVCKIADFGLARVIEDNE 410
P07948|LYN_HUMAN     FSAQIAEGMAYIERKNYIHRDLRAANVLVSESLMCKIADFGLARVIEDNE 396
P06239|LCK_HUMAN     MAAQIAEGMAFIEERNYIHRDLRAANILVSDTLSCKIADFGLARLIEDNE 393
P51451|BLK_HUMAN     MSAQIAEGMAYIERMNSIHRDLRAANILVSEALCCKIADFGLARIIDS-E 388

P12931|SRC_HUMAN     YTARQGAKFPIKWTAPEAALYGRFTIKSDVWSFGILLTELTTKGRVPYPG 468 Y419(autophosphorylation)
P07947|YES_HUMAN     YTARQGAKFPIKWTAPEAALYGRFTIKSDVWSFGILQTELVTKGRVPYPG 475 Y426(autophosphorylation)
P09769|FGR_HUMAN     YNPCQGSFKPIKWTAPEAALFGRFTIKSDVWSFGILLTELITKGRIPYPG 461 Y412(autophosphorylation)
P06241|FYN_HUMAN     YTARQGAKFPIKWTAPEAALYGRFTIKSDVWSFGILLTELVTKGRVPYPG 469 Y420(autophosphorylation)
P08631|HCK_HUMAN     YTAREGAKFPIKWTAPEAINFGSFTIKSDVWSFGILLMEIVTYGRIPYPG 460 Y411(autophosphorylation)
P07948|LYN_HUMAN     YTAREGAKFPIKWTAPEAINFGCFTIKSDVWSFGILLYEIVTYGKIPYPG 446 Y397(autophosphorylation)
P06239|LCK_HUMAN     YTAREGAKFPIKWTAPEAINYGTFTIKSDVWSFGILLTEIVTHGRIPYPG 443 Y394(autophosphorylation)
P51451|BLK_HUMAN     YTAQEGAKFPIKWTAPEAIHFGVFTIKADVWSFGVLLMEVVTYGRVPYPG 438 Y389(autophosphorylation)

P12931|SRC_HUMAN     MVNREVLDQVERGYRMPCPPECPESLHD-LMCQCWRKEPEERPTFEYLQA 517
P07947|YES_HUMAN     MVNREVLEQVERGYRMPCPQGCPESLHE-LMNLCWKKDPDERPTFEYIQS 524
P09769|FGR_HUMAN     MNKREVLEQVEQGYHMPCPPGCPASLYE-AMEQTWRLDPEERPTFEYLQS 510
P06241|FYN_HUMAN     MNNREVLEQVERGYRMPCPQDCPISLHE-LMIHCWKKDPEERPTFEYLQS 518
P08631|HCK_HUMAN     MSNPEVIRALERGYRMPRPENCPEELYN-IMMRCWKNRPEERPTFEYIQS 509
P07948|LYN_HUMAN     RTNADVMTALSQGYRMPRVENCPDELYD-IMKMCWKEKAEERPTFDYLQS 495
P06239|LCK_HUMAN     MTNPEVIQNLERGYRMVRPDNCPEELYQ-LMRLCWKERPEDRPTFDYLRS 492
P51451|BLK_HUMAN     MSNPEVIRNLERGYRMPRPDTCPPELYRGVIAECWRSRPEERPTFEFLQS 488

P12931|SRC_HUMAN     FLEDYFTSTEPQYQPGENL 536 Y530(Phosphorulated by CSK)
P07947|YES_HUMAN     FLEDYFTATEPQYQPGENL 543 Y537(Phosphorulated by CSK)
P09769|FGR_HUMAN     FLEDYFTSAEPQYQPGDQT 529 Y523(Phosphorulated by CSK)
P06241|FYN_HUMAN     FLEDYFTATEPQYQPGENL 537 Y531(Phosphorulated by CSK)
P08631|HCK_HUMAN     VLDDFYTATESQYQQQP-- 526 Y522(Phosphorulated by CSK)
P07948|LYN_HUMAN     VLDDFYTATEGQYQQQP-- 512 Y508(Phosphorulated by CSK)
P06239|LCK_HUMAN     VLEDFFTATEGQYQPQP-- 509 Y505(Phosphorulated by CSK)
P51451|BLK_HUMAN     VLEDFYTATERQYELQP-- 505 Y501(Phosphorulated by CSK)
```

Supplementary Table S1. DNA oligomers used for plasmid construction.

Oligomer name	Sequence (5′-3′)
Srctide_F	gggatccccggaattc**ggcgaagaaccgctgtattggagctttccggcgaaaaaaaaa**taa tcgagcggccgcatc (EcoRI, **Srctide**, stop codon, ΔXhoI)
Srctide_R	gatgcggccgctcga tta**ttttttttcgccggaaagctccaatacagcggttcttcgcc**gaattccggggatccc (ΔXhoI, stop codon, **Srctide**, EcoRI)
pGEX-6P-1_EcoRI	gaattccggggatcccaggg (EcoRI)
pGEX-6P-1_ΔXhoI	tcgagcggccgcatcgtgac (ΔXhoI)
GST Srctide_pCDF1b_F	caagagtccggatcc**atgtcccctatactaggtta** (BamHI, **GST**)
GSTSrctide_pCDF1b_R	ttctttaccagactc**ttattttttttggcgaacg** (XhoI, **Srctide**)
pCDF1b_BamHI	ggatccggactcttgtcgtc (BamHI)
pCDF1b_XhoI	gagtctggtaaagaaaccgc (XhoI)
pET21a_ Src_F	atgggtcgcggatccatgggtagcaacaagagc (BamHI, start codon)
pET21a_Src_ R	gtggtggtgctcgaggaggttctccccgggctg (XhoI)
pET21a_ Fgr_F	atgggtcgcggatccatgggctgtgtgttctgc (BamHI, start codon)
pET21a_ Fgr_R	gtggtggtgctcgagtgtctgatccccgggctg (XhoI)
pET21a_Hck F	atgggtcgcggatccatgggctgtgtgttctgc (BamHI, start codon)
pET21a_Hck R	gtggtggtgctcgagtggctgctgttggtactg (XhoI)
pET21a_ Yes_F	atgggtcgcggatccatgggctgcattaaaagt (BamHI, start codon)
pET21a_ Yes_R	gtggtggtgctcgagtaaattttctcctggctg (XhoI)
pET21a_Blk_F	atgggtcgcggatccatggggctggtaagtagc (BamHI, start codon)
pET21a_ Blk_R	gtggtggtgctcgaggggctgcagctcgtactg (XhoI)
pET21a_ Fyn_F	atgggtcgcggatccatgggctgtgtgcaatgt (BamHI, start codon)
pET21a_Fyn_ R	gtggtggtgctcgagcaggttttcaccaggttg (XhoI)
pET21a_ Lck_F	atgggtcgcggatccatgggctgtggctgcagc (BamHI, start codon)
pET21a_ Lck_R	gtggtggtgctcgagaggctgaggctggtactg (XhoI)
pET21a_ Lyn_F	atgggtcgcggatccatgggatgtataaaatca (BamHI, start codon)
pET21a_ Lyn_R	gtggtggtgctcgagaggctgctgctggtattg (XhoI)
pET21a_BamHI	ggatccgcgacccatttgct (BamHI)
pET21a_XhoI	ctcgagcaccaccaccacca (XhoI)
pcDNA3.1_Src_F	gctggatatctgcagaattcatgggtagcaacaagagcaag (EcoRI, start codon)
pcDNA3.1_Src_R	ttggtaccgagctcggatcctca**cttatcgtcgtcatccttgtaatc**gaggttctccccgggctg (BamHI, stop codon, **FLAG**)
pcDNA3.1_Lck_F	gctggatatctgcagaattcatgggctgtggctgcagctca (EcoRI, start codon)
pcDNA3.1_Lck_R	ttggtaccgagctcggatcctca**cttatcgtcgtcatccttgtaatc**aggctgaggctggtactg (BamHI, stop codon, **FLAG**)
pcDNA3.1_Hck_F	gctggatatctgcagaattcatgggggggcgctcaagctgc (EcoRI, start codon)
pcDNA3.1_Hck_R	ttggtaccgagctcggatcctca**cttatcgtcgtcatccttgtaatc**tggctgctgttggtactg

	(BamHI, stop codon, **FLAG**)
pcDNA3.1_Blk_F	gctggatatctgcagaattcatggggctggtaagtagcaaa (EcoRI, start codon)
pcDNA3.1_Blk _R	ttggtaccgagctcggatcctca**cttatcgtcgtcatccttgtaatc**gggctgcagctcgtactg (BamHI, stop codon, **FLAG**)
pcDNA3.1_ BamHI	ggatccgagctcggtaccaa (BamHI)
pcDNA3.1_EcoRI	gaattctgcagatatccagc (EcoRI)
pHEK293_Yes_F	ctccccgggctcgagggatccatgggctgcattaaaagtaaa (BamHI, start codon)
pHEK293_Yes_R	tgcctgcaggtcgactctagatta**cttatcgtcgtcatccttgtaatc**taaattttctcctggctg (XbaI, stop codon, **FLAG**)
pHEK293_Fyn_F	ctccccgggctcgagggatccatgggctgtgtgcaatgtaag (BamHI, start codon)
pHEK293_Fyn_R	tgcctgcaggtcgactctagatta**cttatcgtcgtcatccttgtaatc**caggttttcaccaggttg (XbaI, stop codon, **FLAG**)
pHEK293_Lyn_F	ctccccgggctcgagggatccatgggatgtataaaatcaaaa (BamHI, start codon)
pHEK293_Lyn_R	tgcctgcaggtcgactctagatta**cttatcgtcgtcatccttgtaatc**aggctgctgctggtattg (XbaI, stop codon, **FLAG**)
pHEK293_Fgr_F	ctccccgggctcgagggatccatgggctgtgtgttctgcaag (BamHI, start codon)
pHEK293_Fgr_R	tgcctgcaggtcgactctagatta**cttatcgtcgtcatccttgtaatc**tgtctgatccccgggctg (XbaI, stop codon, **FLAG**)
pHEK293_BamHI	ggatccctcgagcccggggag (BamHI)
pHEK293_XbaI	tctagagtcgacctgcaggca (XbaI)
pSP64 poly(A)_ Src_F	aagcttgggctgcagatgggtagcaacaagagc (PstI, start codon)
pSP64 poly(A)_Src_ R	cggggatcctctagatta**cttatcgtcgtcatccttgtaatc**gaggttctccccgggctg (XbaI, stop codon,**FLAG**)
pSP64 poly(A)_ Fgr_F	aagcttgggctgcagatgggctgtgtgttctgc (PstI, start codon)
pSP64 poly(A)_ Fgr_R	cggggatcctctagatta**cttatcgtcgtcatccttgtaatc**tgtctgatccccgggctg (XbaI, stop codon, **FLAG**)
pSP64 poly(A)_Hck F	aagcttgggctgcagatgaagtccaagttcctc (PstI, start codon)
pSP64 poly(A)_Hck R	cggggatcctctagatta**cttatcgtcgtcatccttgtaatc**tggctgctgttggtactg (XbaI, stop codon, **FLAG**)
pSP64 poly(A)_ Yes_F	aagcttgggctgcagatgggctgcattaaaagt (PstI, start codon)
pSP64 poly(A)_ Yes_R	cggggatcctctagatta**cttatcgtcgtcatccttgtaatc**taaattttctcctggctg (XbaI, stop codon, **FLAG**)
pSP64 poly(A)_Blk_F	aagcttgggctgcagatggggctggtaagtagc (PstI, start codon)
pSP64 poly(A)_ Blk_R	cggggatcctctagatta**cttatcgtcgtcatccttgtaatc**gggctgcagctcgtactg (XbaI, stop codon, **FLAG**)
pSP64 poly(A)_ Fyn_F	aagcttgggctgcagatgggctgtgtgcaatgt (PstI, start codon)
pSP64 poly(A)_Fyn_ R	cggggatcctctagatta**cttatcgtcgtcatccttgtaatc**caggttttcaccaggttg (XbaI, stop codon, **FLAG**)
pSP64 poly(A)_ Lck_F	aagcttgggctgcagatgggctgtggctgcagc (PstI, start codon)
pSP64 poly(A)_ Lck_R	cggggatcctctagatta**cttatcgtcgtcatccttgtaatc**aggctgaggctggtactg (XbaI, stop codon, **FLAG**)
pSP64 poly(A)_ Lyn_F	aagcttgggctgcagatgggatgtataaaatca (PstI, start codon)
pSP64 poly(A)_ Lyn_R	cggggatcctctagatta**cttatcgtcgtcatccttgtaatc**aggctgctgctggtattg (XbaI, stop codon, **FLAG**)
pSP64 poly(A)_ PstI	ctgcagcccaagcttgtattc (PstI)
pSP64 poly(A)_XbaI	tctagaggatccccgggcgag (XbaI)
pF25A ICE T7_ Src_F	tataaagcgatcgccatgggtagcaacaagagc (SgfI, start codon)
pF25A ICE T7_Src_ R	ccgaattcgtttaaac**cttatcgtcgtcatccttgtaatc**gaggttctccccgggctg (PmeI, stop codon,**FLAG**)
pF25A ICE T7_ Fgr_F	tataaagcgatcgccatgggctgtgtgttctgc (SgfI, start codon)

pF25A ICE T7_ Fgr_R	ccgaattcgtttaaaccttatcgtcgtcatccttgtaatctgtctgatccccgggctg (PmeI, stop codon,FLAG)
pF25A ICE T7_Hck F	tataaagcgatcgccatgaagtccaagttcctc (SgfI, start codon)
pF25A ICE T7_Hck R	ccgaattcgtttaaaccttatcgtcgtcatccttgtaatctggctgctgttggtactg (PmeI, stop codon,FLAG)
pF25A ICE T7_ Yes_F	tataaagcgatcgccatgggctgcattaaaagt (SgfI, start codon)
pF25A ICE T7_ Yes_R	ccgaattcgtttaaaccttatcgtcgtcatccttgtaatctaaattttctcctggctg (PmeI, stop codon,FLAG)
pF25A ICE T7_Blk_F	tataaagcgatcgccatggggctggtaagtagc (SgfI, start codon)
pF25A ICE T7_ Blk_R	ccgaattcgtttaaaccttatcgtcgtcatccttgtaatcgggctgcagctcgtactg (PmeI, stop codon,FLAG)
pF25A ICE T7_ Fyn_F	tataaagcgatcgccatgggctgtgtgcaatgt (SgfI, start codon)
pF25A ICE T7_Fyn_ R	ccgaattcgtttaaaccttatcgtcgtcatccttgtaatccaggttttcaccaggttg (PmeI, stop codon,FLAG)
pF25A ICE T7_ Lck_F	tataaagcgatcgccatgggctgtggctgcagc (SgfI, start codon)
pF25A ICE T7_ Lck_R	ccgaattcgtttaaaccttatcgtcgtcatccttgtaatcaggctgaggctggtactg (PmeI, stop codon,FLAG)
pF25A ICE T7_ Lyn_F	tataaagcgatcgccatgggatgtataaaatca(SgfI, start codon)
pF25A ICE T7_ Lyn_R	ccgaattcgtttaaaccttatcgtcgtcatccttgtaatcaggctgctgctggtattg (PmeI, stop codon,FLAG)
pF25A ICE T7_ PstI	ggcgatcgctttatattttatttt (SgfI)
pF25A ICE T7_XbaI	gtttaaacgaattcgggctcggt (PmeI)
pFQE30_ Src_F	caccatcacggatccatgggtagcaacaagagc(BamHI, start codon)
pFQE30_Src_ R	cagctaattaagcttttacttatcgtcgtcatccttgtaatcgaggttctccccgggctg(HindIII, stop codon,FLAG)
pFQE30_ Fgr_F	caccatcacggatccatgggctgtgtgttctgc(BamHI, start codon)
pFQE30_ Fgr_R	cagctaattaagcttttacttatcgtcgtcatccttgtaatctgtctgatccccgggctg(HindIII, stop codon,FLAG)
pFQE30_Hck F	caccatcacggatccatgaagtccaagttcctc(BamHI, start codon)
pFQE30_Hck R	cagctaattaagcttttacttatcgtcgtcatccttgtaatctggctgctgttggtactg(HindIII, stop codon,FLAG)
pFQE30_ Yes_F	caccatcacggatccatgggctgcattaaaagt(BamHI, start codon)
pFQE30_ Yes_R	cagctaattaagcttttacttatcgtcgtcatccttgtaatctaaattttctcctggctg(HindIII, stop codon,FLAG)
pFQE30_Blk_F	caccatcacggatccatggggctggtaagtagc(BamHI, start codon)
pFQE30_ Blk_R	cagctaattaagcttttacttatcgtcgtcatccttgtaatcgggctgcagctcgtactg(HindIII, stop codon,FLAG)
pFQE30_ Fyn_F	caccatcacggatccatgggctgtgtgcaatgt(BamHI, start codon)
pFQE30_Fyn_ R	cagctaattaagcttttacttatcgtcgtcatccttgtaatccaggttttcaccaggttg(HindIII, stop codon,FLAG)
pFQE30_ Lck_F	caccatcacggatccatgggctgtggctgcagc(BamHI, start codon)
pFQE30_ Lck_R	cagctaattaagcttttacttatcgtcgtcatccttgtaatcaggctgaggctggtactg(HindIII, stop codon,FLAG)
pFQE30_ Lyn_F	caccatcacggatccatgggatgtataaaatca(BamHI, start codon)
pFQE30_ Lyn_R	cagctaattaagcttttacttatcgtcgtcatccttgtaatcaggctgctgctggtattg(HindIII, stop codon,FLAG)
pFQE30_ BamHI	ggatccgtgatggtgatggtg (BamHI)
pFQE30_HindIII	aagcttaattagctgagcttg (HindIII)
Src_Y419F_F	attgaagacaatgagttcacggcgcggcaaggt
Src_Y419F_R	accttgccgcgccgtgaactcattgtcttcaat
Fgr_Y412_F	atcaaggacgatgagttcaacccctgccaaggt
Fgr_Y412_R	accttggcaggggttgaactcatcgtccttgat
Hck_Y411F_F	attgaggacaacgagttcacggctcgggaaggg
Hck_Y411F_R	cccttcccgagccgtgaactcgttgtcctcaat
Yes_Y426F_F	attgaagacaatgaattcacagcaagacaaggt
Yes_Y426F_R	accttgtcttgctgtgaattcattgtcttcaat
Blk_Y389F_F	atcatcgacagtgaattcacggcccaagagggg

Blk_Y389F_R	cccctcttgggccgtgaattcactgtcgatgat
Fyn_Y365F_F	atagaagacaatgagttcacagcaagacaaggt
Fyn_Y365F_R	accttgtcttgctgtgaactcattgtcttctat
Lck_Y424F_F	attgaggacaacgagttcacagccagggagggg
Lck_Y424F_R	cccctccctggctgtgaactcgttgtcctcaat
Lyn_Y397F_F	attgaagataatgagttcacagcaagggaagg
Lyn_Y397F_R	ccttcccttgctgtgaactcattatcttcaat

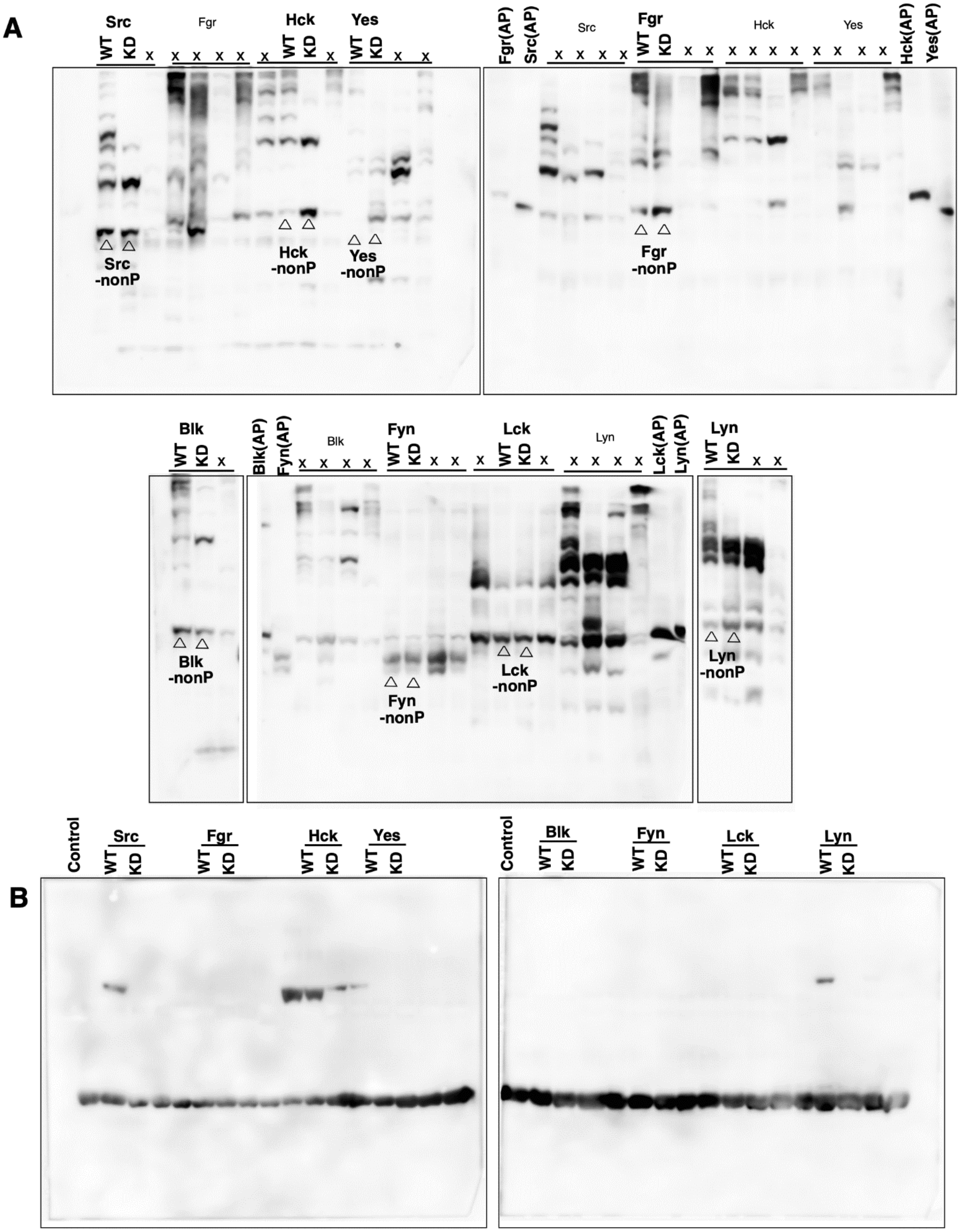

Supplementary Figure S1. Raw images used for Fig. 2. The band position of the unphosphorylated form of the kinase was assigned by the banding pattern of alkaline phosphatase (AP)-treated lysates shown in Fig. S1A. For AP treatment, 293 cells expressing the kinase were lysed with cell lysis buffer [50 mM Tris–HCl (pH8.0), 150 mM NaCl, 0.5%(w/v) sodium deoxycholate, 1.0%(v/v) Nonidet P40] and the soluble fraction of the lysate was incubated with AP in 50 mM Tris–HCl (pH 9.0) and 1 mM $MgCl_2$ for 3 h at 37 °C. The reactions were terminated by adding a half volume of sample-loading buffer for SDS-PAGE, consisting of 195 mM Tris–HCl (pH 6.8), 3.0% w/v SDS, 30% v/v glycerol, 15% v/v 2-sulfanylethanol, and 0.10% w/v bromophenol blue.

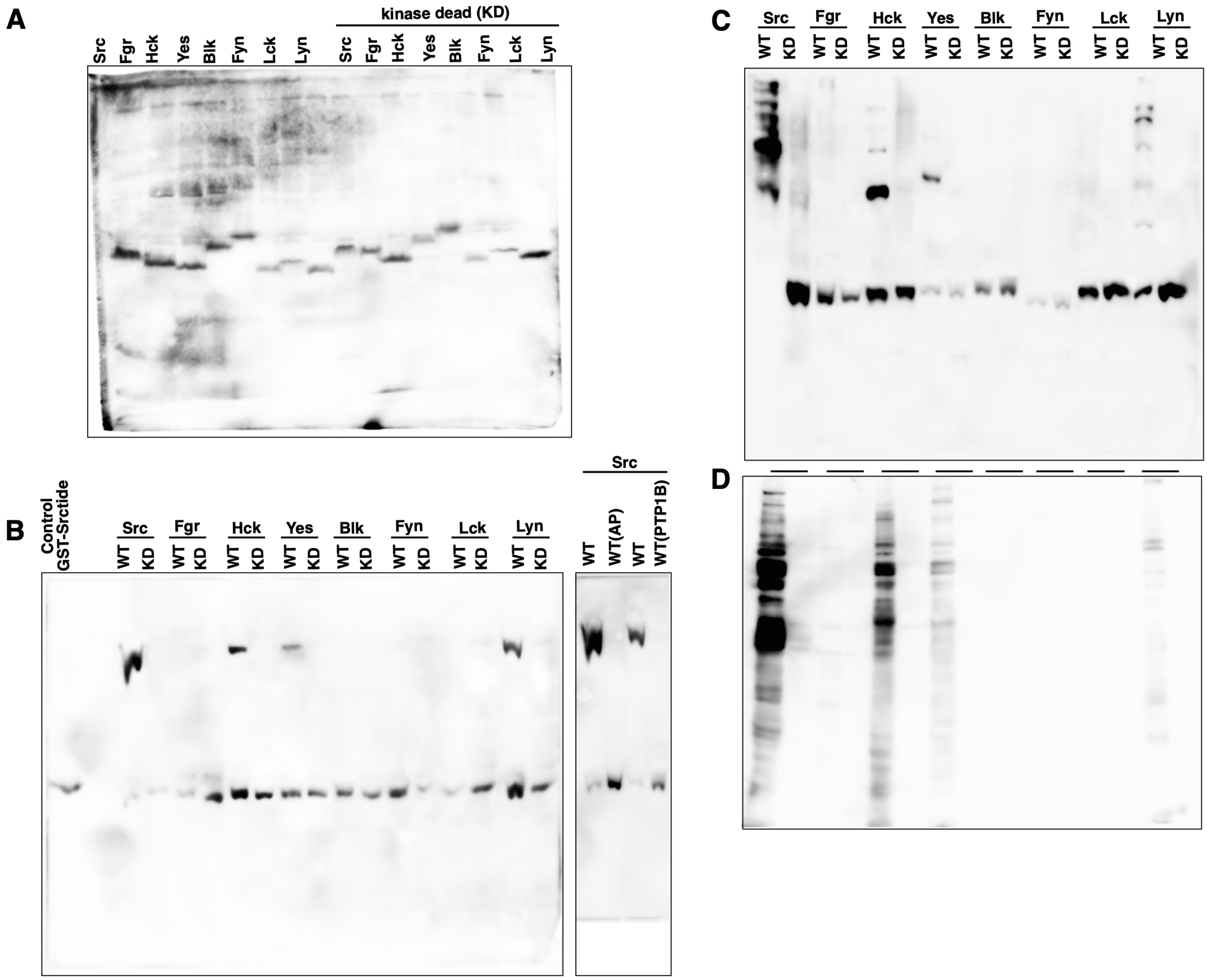

Supplementary Figure S2. Raw images used for Fig. 3. The right panel of Fig. S2B shows alkaline phosphatase (AP) and tyrosine phosphatase (PTP 1B) assays of the Src-expressed lysate. For the AP assay, the lysate was incubated with AP in 50 mM Tris–HCl (pH 9.0) and 1 mM $MgCl_2$ for 3 h at 37 °C. For the PTP 1B assay, the lysate was incubated with PTP1B in 10 mM Tris–HCl (pH 8.0) and 50 mM NaCl, 1 mM dithiothreitol, and 1 mM $MnCl_2$ for 3 h at 37 °C. The reactions were terminated by adding a half volume of sample-loading buffer for SDS-PAGE, consisting of 195 mM Tris–HCl (pH 6.8), 3.0% w/v SDS, 30% v/v glycerol, 15% v/v 2-sulfanylethanol, and 0.10% w/v bromophenol blue. The reactions were analyzed by Phos-tag SDS-PAGE (10% w/v polyacrylamide, 20 μM Zn^{2+}–Phos-tag), followed by Western blotting with anti-GST antibody.

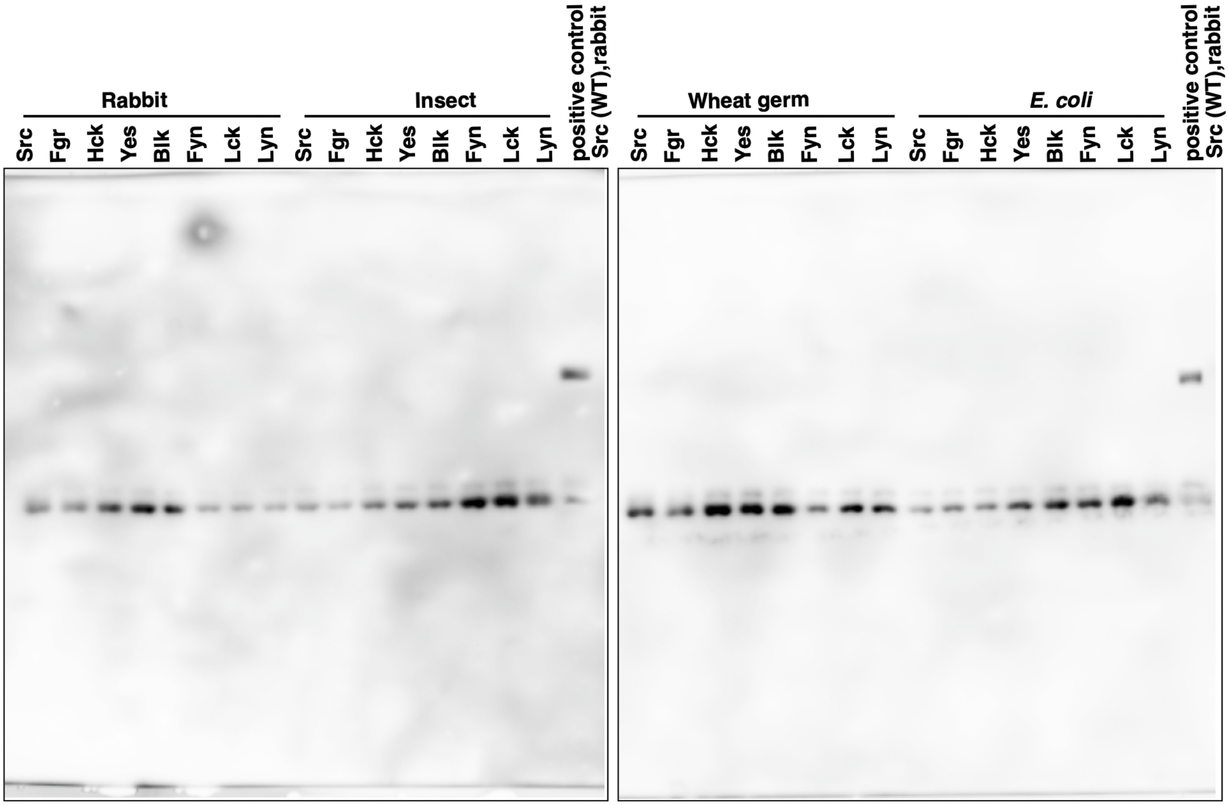

Supplementary Figure S3. In vitro GST-Srctide phosphorylation assay of kinase-dead mutant of SFKs. Kinase-dead mutant of each SFK expressed by each cell-free protein expression system was purified by immunoprecipitation with anti-FLAG antibody-bound magnetic beads and then subjected to in vitro GST-Srctide phosphorylation assay. Reactions were applied to Phos-tag SDS-PAGE gels (10% w/v polyacrylamide, 20 μM Zn^{2+}–Phos-tag). The gels were analyzed by Western blotting with anti-GST antibody.

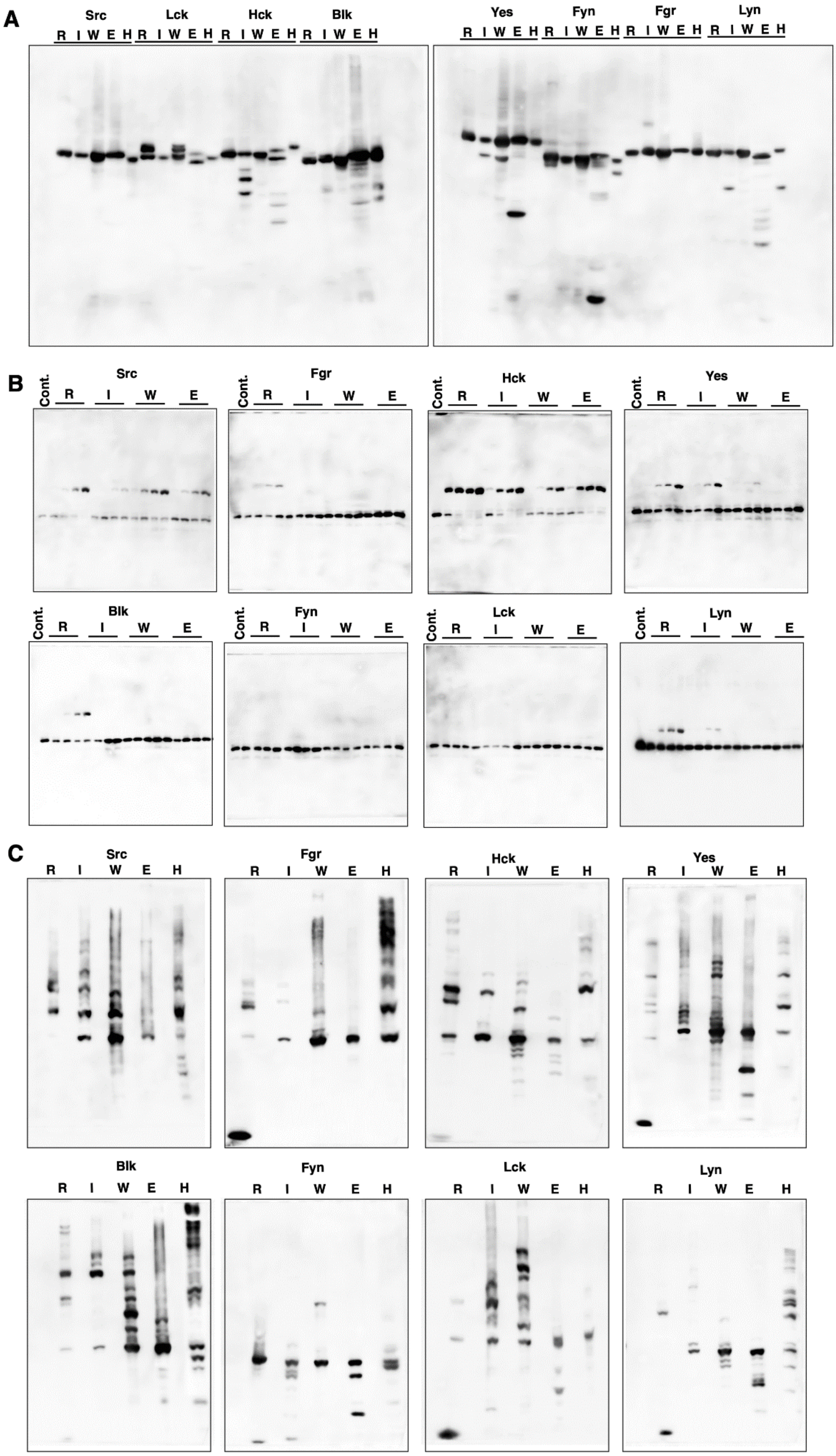

Supplementary Figure S4. Raw images used for Fig. 4.

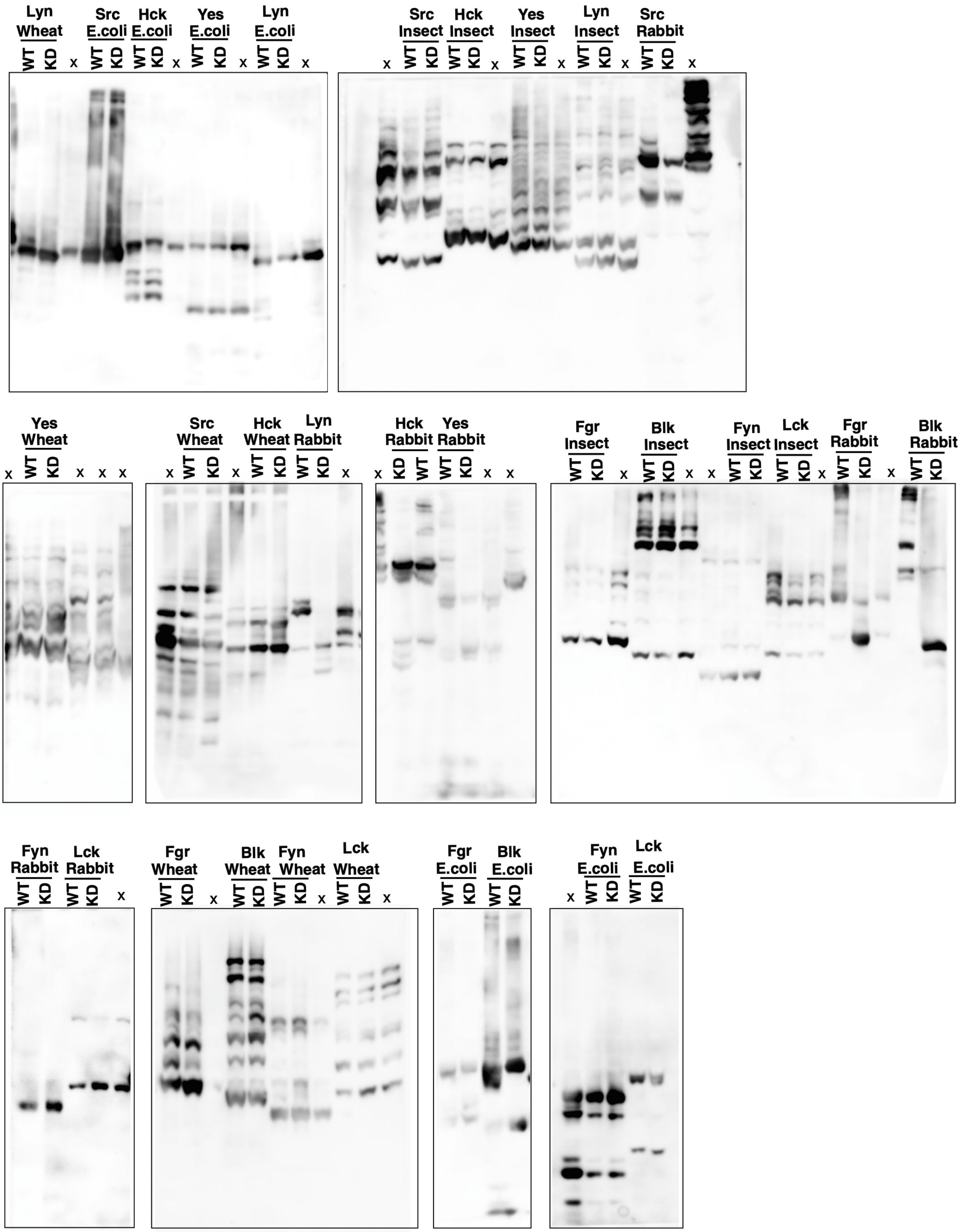

Supplementary Figure S5. Raw images used for Fig. 5.

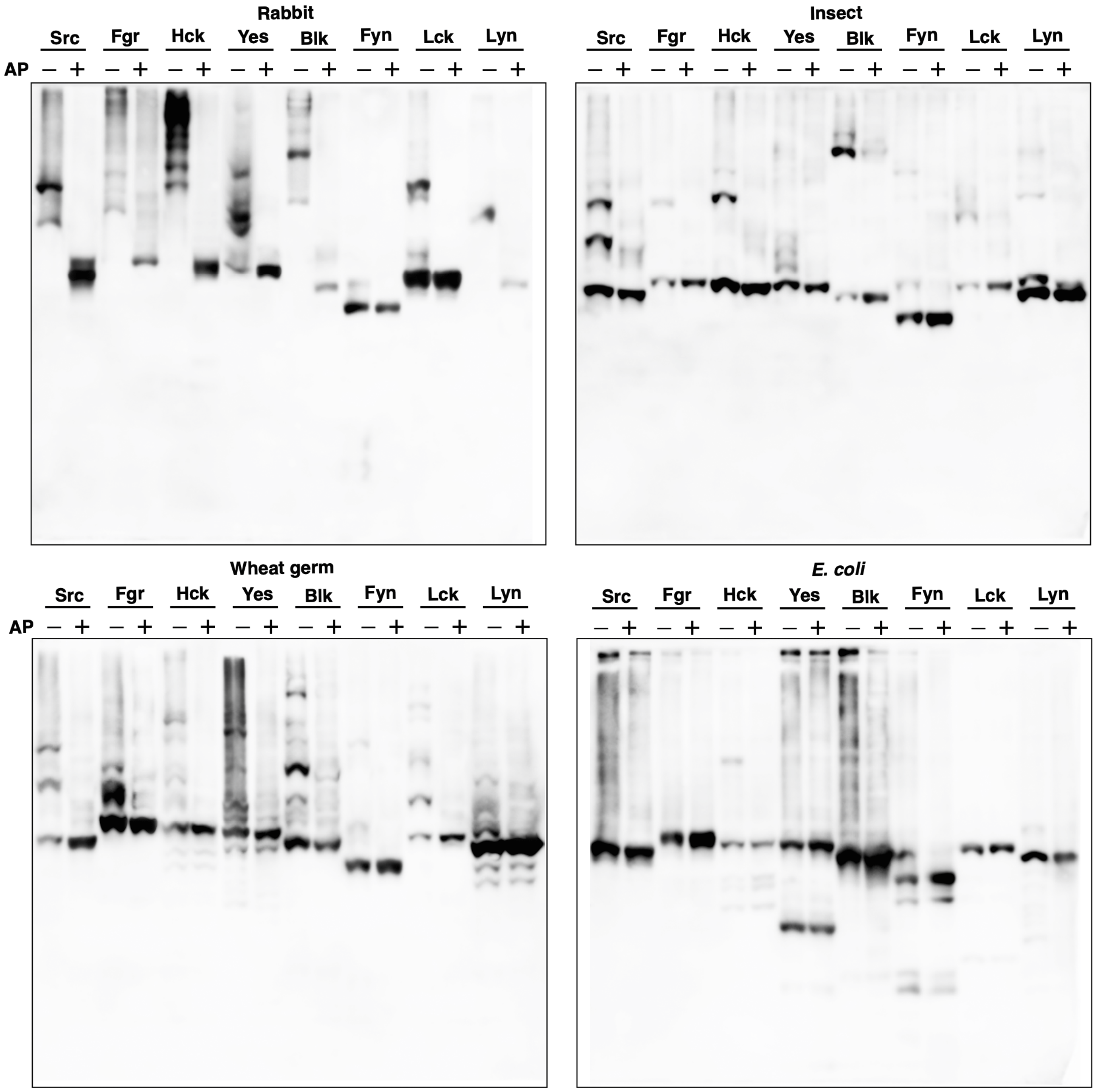

Supplementary Figure S6. Alkaline phosphatase (AP) assay of SFKs expressed by four types of cell-free expression systems; TnT SP6 Quick Coupled Transcription/Translation System (rabbit reticulocyte lysate system), TnT T7 Insect Cell Extract Protein Expression System, TnT SP6 High-Yield Wheat Germ Protein Expression System, S30 T7 High-Yield Protein Expression System (*E. coli* lysate system). For the AP assay, the protein expression reaction solution was incubated with AP in 50 mM Tris–HCl (pH 9.0) and 1 mM $MgCl_2$ for 3 h at 37 °C. The reactions were terminated by adding a half volume of sample-loading buffer for SDS-PAGE, consisting of 195 mM Tris–HCl (pH 6.8), 3.0% w/v SDS, 30% v/v glycerol, 15% v/v 2-sulfanylethanol, and 0.10% w/v bromophenol blue. The reactions were analyzed by Phos-tag SDS-PAGE (7% w/v polyacrylamide, 20 μM Zn^{2+}–Phos-tag), followed by Western blotting with anti-FLAG antibody.
